# A standardized in vitro bioengineered skin for penetrating wound modeling

**DOI:** 10.1007/s44164-025-00082-x

**Published:** 2025-02-21

**Authors:** C. Sofia Salazar Silva, Werner Petzold, Ulrike Hirsch, Christian E. H. Schmelzer, Andrea Friedmann

**Affiliations:** 1 Fraunhofer Institute for Microstructure of Materials and Systems IMWS, Walter-Huelse-Strasse 1, 06120 Halle (Saale), Germany; 2 Institute of Pharmacy, Faculty of Natural Sciences I, Martin Luther University Halle-Wittenberg, 06120 Halle (Saale), Germany

**Keywords:** Tissue engineering, Biomaterials, Epidermal barrier, Collagen, Stamping needle

## Abstract

Over the years, significant progress has been made in developing cost-effective and ethical in vitro bioengineered skin substitutes to study cutaneous wound healing processes. Rodents and small animal models are not optimal due to physiological differences in their skin compared to human skin. The generation of reproducible and precise wounds is key to obtaining comparable results. We created a three-dimensional skin wounding model by growing a fully differentiated, stratified squamous epithelium from human keratinocytes at an air–liquid interface on a type I collagen scaffold concealed with human dermal BJ fibroblasts. To generate the wounds, a stamp with incorporated needles with a length of 250 µm was used to puncture the epidermis to produce standardized wounds. The stamping needle technique is a practical and inexpensive method for creating length-tailored wounds on three-dimensional skin models. The effectiveness of this technique in treating 3D skin models was demonstrated, accompanied by an evaluation of the model’s functionality in terms of cell proliferation, differentiation, and immunological characteristics.

## Introduction

The utilization of in vitro bioengineered skin models presents an invaluable approach to the study of critical aspects of skin biology. These models, comprising epidermal keratinocytes cultivated on dermal equivalents, whether natural or synthetic scaffolds seeded with fibroblasts, mimic the complex structure of the skin. Their use has led to effective results in enhancing the skin wound healing process in vitro (for review, see [1]). However, the applicability of 3D skin wound models in research is limited due to their poor reproducibility and low cost-effectiveness.

Recent advancements in understanding the biological mechanisms underlying skin and wound repair have led to significant progress in wound treatment approaches. Traditionally, animal models have served as the standard for investigating wound healing. However, using small animal models, such as rodents, poses challenges due to their biological factors like strain, age, microbiome composition, and species-specific variations [2–4]. Moreover, the associated expenses of animal housing, veterinary care, logistics, and ethical implications further increasingly limit their suitability for such studies. Hence, there is a pressing need for cost-effective and reliable alternatives to propel advancements in in vitro testing within the bioengineering field.

The ultimate goal of a bioengineered skin model is to resemble the anatomical and physiological characteristics and functions of human skin. These properties are necessary to replicate normal and pathological skin conditions efficiently. In addition, the reliability of such models is essential for application in toxicological, pharmaceutical, and large-scale research, such as conducting high-throughput screening studies [5]. The classification of bioengineered skin substitutes depends on their intended application, which can vary between therapeutic and research purposes. The latter includes the use of bioengineered 3D skin systems for exploring fundamental mechanisms in skin biology, including interactions of growth factors, wound healing processes, and the pathology of dermatological diseases [6]. Although these skin models are simple to produce, they do not meet regulatory requirements for therapeutic skin substitutes. Therefore, ensuring quality management and reproducibility of these products is critical. Bioengineered skin models designed for therapeutic applications serve as valuable toxicological systems for evaluating products that come into contact with human skin. The skin models are used to test corrosivity in cell viability upon exposure to cosmetics, household products, or pharmaceuticals [6]. The irritancy and toxicity are measured by the release of inflammatory cytokines such as IL-1α [7].

An essential factor in creating bioengineered skin in tissue engineering is the fabrication of scaffolds that provide structural support and mimic the extracellular matrix (ECM). To achieve this, the materials used should include outstanding biocompatibility, appropriate microstructure, manipulable biodegradability, and good mechanical properties [8]. Collagen meets all these requirements, making it one of the most used scaffold materials in tissue engineering. Mainly, collagen scaffolds promote cell and tissue attachment, proliferation, and migration [9]. This material can be manipulated to improve scaffolds’ mechanical and physical properties using combinable cross-linking agents [10].

It is well established that cells cultured in 3D exhibit different morphology, migration, molecule signaling, and metabolic functions than 2D-cultured cells [11]. In vivo, cells in the ECM migrate in all directions. This situation is, therefore, better simulated by a 3D cell culture to transfer the in vitro wounding model to a 3D assay. The 3D wounding assays are primarily bioengineered skin models developed to observe the healing of chronic or acute wounds. Examples of skin models for dermatological wound healing testing are Apligraf, Hyalograft 3D, and TissueTech Autograft System [12]. The skin wound constructs comprise a bilayer structure that mimics the natural skin components. The dermis is made of a matrix such as collagen scaffolds, synthetic polymers, or glycosaminoglycans seeded with fibroblasts. Over this dermal layer, keratinocytes are cultivated to form the epidermis. The cells used for the epidermal layer can be either immortalized cell lines or primary cells. During the air-lifting phase, the keratinocytes seeded on the top layer undergo a process where they lose their nuclei, closely resembling the properties of natural skin. Once the 3D skin model construct has matured, controlled wounding is induced through various methods, such as mechanical or chemical means. These methods may involve scratching, cutting, puncturing, or applying specific substances to simulate different types of wounds [13]. Typical instruments used include needles and scalpels, along with techniques such as abrasion or puncture. Thermal wounding uses heated tools or electrical cauterization. The poor reproducibility or complete detachment of the epidermis due to mechanical or thermal wounding is a disadvantage [14]. Therefore, current approaches focus on improving a bioengineered skin wound model.

Using an in vitro wound model, the cellular crosstalk and development of fibroblasts and keratinocytes can be studied. The 3D skin wound model allows gaining knowledge about cell migration and interaction in the wound healing process. Furthermore, it is vital for comprehending tissue regeneration in response to various factors, including pharmacological agents, skincare products, cosmetic testing, and therapeutic agents. The generation of a wound model is challenging due to its standardization and reproducibility between multiple tests. The injury’s size, depth, and shape can vary between studies and impair the data quality [14]. Another standardized wounding model uses lasers because of their reproducibility and precision in inducing wounds. However, a drawback of this method is that the wound produced by the laser can simulate unwanted burn injuries [15]. The heat generated by the laser can induce premature platelet aggregation, protein denaturation, or vessel constriction, ultimately leading to the formation of necrotic tissue [16]. Further research is required to comprehend how to attain precise and clean perforation of the epidermal layer for bioengineered skin wound models. This would assist in developing dependable models for investigating wound healing, which would be helpful in testing new treatments and identifying the mechanisms involved in wound healing.

The purpose of this study is to develop an innovative and reproducible approach for creating standardized epidermal abrasion wound models on 3D human skin equivalents using two approaches: microneedle-stamping and phenol at varying concentrations. This leverages the advantages of both techniques: microneedle-stamping for generating defined and precise wounds under sterile conditions and phenol for controlled obliteration of the epidermis with regulated depth and area. We aim to create a practical, inexpensive, and rapid method for providing a functional in vitro model of human wound healing. To achieve this, we will assess the morphology, viability, and immunological function of the wounded tissue as a mechanical barrier, using established methods such as histological staining, metabolic assays, and cytokine expression analysis.

## Results and discussion

### Cell viability

**Fig. 1:**
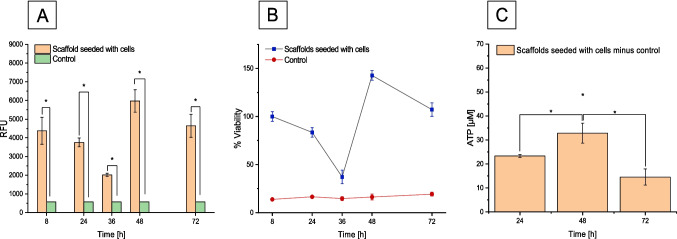
**A** The relative ability of BJ fibroblast cells types to reduce resazurin to resorufin data showed in RFU. **B** The amount of fluorescence produced is proportional to the percentage of living cells: negative control scaffold w/o cells and Cell Titer-Blue mixture for blank. **C** ATP concentration calculated from the standard curve minus the control (scaffold without cells). For each cell viability test, six scaffolds were seeded with BJ cells and two scaffolds without cells were used for the control. Data are mean ± SD in triplicate. Values significantly different (*p < 0.01, Bonferroni) from control

To measure the metabolic activity as an indicator of cell viability of BJ fibroblasts seeded on collagen scaffolds, the Cell Titer Blue Cell Viability Assay was used. The relative fluorescence unit (RFU) was recorded for cells initially inoculated at a number of 1.3 × 10^5^ cells per scaffold, passage 38 on *t* = 8 h (Fig. 1A). The fluorescence intensity is proportional to the percentage of viable cells at different time points (Fig. 1B). Collagen scaffolds not seeded with cells were used as control. The RFU detected on the control collagen scaffolds was not significant. The results show that the cells seeded on the collagen scaffold retain viability as they continuously reduce resazurin to resorufin (Fig. 1A). Full cell viability, indicated by 100%, was achieved 8 h after inoculating the scaffold with the BJ fibroblasts. After 24 h of incubation, the recorded cell viability was 80%. The scaffolds with seeded cells and controls were transferred from a 12-well plate to a 6-well plate for the generation of the 3D skin models. Following this, the lowest viability was recorded 36 h after seeding, with a value of 40% (Fig. 1B).

The reason for this phenomenon may be attributed to the transfer of the samples to a larger environment, resulting in the detachment of several cells that were adhered to the previous well plate. At 48 h after cell seeding on scaffolds, the highest viability of 140% was measured. The data suggest that collagen 3D scaffolds provide a suitable environment for BJ fibroblast cell proliferation. This may be due to the cells adapting to their expanded environment, benefitting from the fresh nutrients provided by a medium change. The cell viability experiment was carried out twice in triplicates under the same parameters, and this low viability was consistently observed.

Another cell viability assay specific for 3D cultures is the CellTiter-Glo 3D Cell Viability Assay. Based on ATP quantitation from metabolically active cells, the assay can determine the number of viable cells in 3D cell culture. The ATP assay is formulated with a solid lytic capacity to assess the intracellular ATP in microtissues. For this test, ATP is directly proportional to the number of viable cells present in the culture medium after cell lysis (Fig. 1C). Following a 24-h scaffold inoculation, the cells had a concentration of 23 µm ATP, and this concentration incremented by 41% with 32-µm ATP after 48 h and hence cell viability. It was observed that the ATP concentration significantly diminished by 52% after 72 h. When assessing the cell proliferation status ATP levels (expressed in Relative Luminescence Units) in both seeded and non-seeded scaffolds, it is crucial to consider that the cell culture initially originated in a 12-well plate. After 24 h, the cell culture was transferred to a 6-well plate, leading to the absence of cells that had initially adhered to the well plates. This change could potentially impact the observed ATP concentration results at 72 h after incubation. Consequently, it is anticipated that the proliferation rate in the respective scaffolds, as indicated by the ATP levels, may differ.

The viability experiment conducted on cells seeded in the collagen scaffolds played a critical role in confirming the scaffold’s biocompatibility for generating the 3D skin wounded model. This served to demonstrate that the collagen scaffolds are indeed biocompatible, and their 3D structure facilitates cell proliferation and migration, essential processes for tissue repair in wounds.

### Skin wounding model with stamping needles

**Fig. 2:**
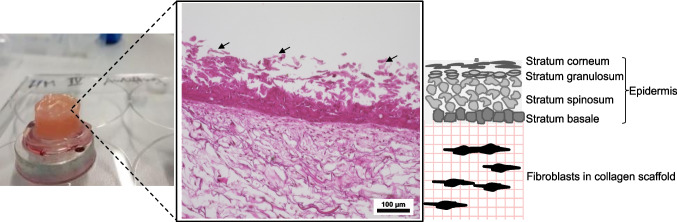
Light microscopic image of H&E staining of the 3D skin model based on collagen scaffold. The intact human skin equivalent serves as the control. Depicted are the three layers of human equivalent skin: the collagen scaffold dermis seeded with fibroblasts, the basal layer of the epidermis with proliferating keratinocytes, and the desquamating corneocytes (indicated by arrows)

The results of the H&E histology analysis revealed that after approximately two weeks of culture at the air–liquid interface, the 3D skin model developed an epidermis consisting of multiple layers of keratinocytes at various stages of differentiation, along with a well-defined *stratum corneum*. Flattened corneocytes reside in the outermost layer and initiate the process of desquamation (Fig. 2, arrows). Furthermore, the developed dermal layer is composed of fibroblasts embedded in a collagen scaffold. The total culture time to create 3D skin models is about 30 days. After this time, the 3D skin models are fully grown and display developed dermal components (Fig. 2).

**Fig. 3:**
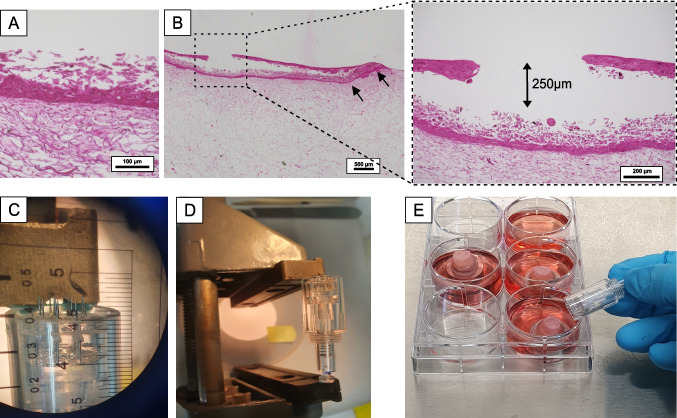
Needle stamping method for wounding and H&E staining. H&E staining of **A** control (no wound) and **B** wounded 3D skin model with stamping needles. The arrows in **B** indicate the attachment of the epidermis to the scaffold’s borders. The needle was adjusted to length to perforate only the epidermis (**C**, **D**). Perforation of the 3D skin models with the microneedles in a sterile environment for further cultivation of the 3D skin models is indicated in **E**

Depicted in Fig. 3A and B is the comparison of the H&E staining between the control (non-wounded) and the wounded 3D skin model, providing evidence of the histological architecture resembling human skin. The use of microneedles for epidermal wounding demonstrated the ability to customize the wound depth to 250 µm, depending on the length of the needles utilized. The wounded tissue exhibits a slight elevation of the epidermal layer but does not display disorganization. Moreover, the epidermis remains attached to the dermis at the scaffold’s borders (Fig. 3B, indicated by arrows), and there is no perforation of the dermal layer by the stamping needles. The wounding procedure proved effective, as evidenced by the elevated position of the samples on the platform comprising the steel ring and Eppendorf tube cap design (Fig. 3D). This elevated platform ensures precise positioning of the 3D skin models and enhances accessibility during the wounding process. The stamping needles implemented were adjusted to the desired wound depth (Fig. 3C). Sharp, sterilized new needles (*n* = 3) were used before each wounding. Thus, it generated clean perforations with the desired depth of 250 µm, rendering it ideal for investigating mechanically induced wounds. This approach proved to be the most advantageous as it offered a quick and inexpensive alternative for creating tailored epidermal wounds. Other authors [17] have used a standardized 3D skin model to show the direct molecular effects of 1-mm-long microneedle penetration on epidermal keratinocytes and dermal fibroblasts after 5 days. The generation of a precise wound was not intended, but the analysis of the gene expression of a wounded bioengineered skin was observed.

Further research groups have also induced mechanical wounds using a biopsy punch, which completely removes the dermal and epidermal layers, resulting in full-thickness wounds on bioengineered skin models [18]. However, these wounds are not defined in terms of localized overstretching and rupture potential. The skin wounding model using stamping needles provides a controlled and reproducible method for studying acute mechanical injuries. This technique creates precise, localized disruptions in the epidermal layer, simulating puncture wounds or minor abrasions. The stamping needle approach allows to examine the immediate cellular responses to mechanical trauma, including the activation of keratinocytes and fibroblasts. It is particularly useful for investigating re-epithelialization processes, as it typically preserves the basement membrane, facilitating the study of keratinocyte migration and proliferation [19]. This model also enables the assessment of early inflammatory responses and the initiation of wound healing cascades, making it valuable for evaluating potential therapeutic interventions for acute wounds.

### Skin wounding model with phenol

**Fig. 4:**
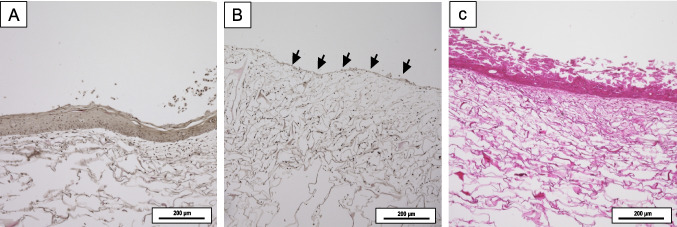
Light microscopic images of H&E staining of the 3D skin model with 5% phenol for 30 s (**A**) and 24 h (**B**). The arrows in **B** indicate the complete detachment of the epidermal layer after necrosis. Control (no wound) in **C**

The second method tested was phenol chemical wounding at different concentrations and incubation times (Table 1), resulting in large wounds that removed the entire epidermal layer (Fig. 4A and B). Phenol is the most important representative of phenolic compounds, characterized by a mono-phenyl ring bonded to a hydroxyl group [20]. It has been used as a non-invasive therapy for skin tumor removal in elderly patients. In dermatological treatment, the application of 100% phenol produces a uniform wound with the objective of stimulating re-epithelialization, rendering it a highly effective chemical peeling [21]. The present study demonstrated that after 24 h post 5% phenol treatment for 30 s, the epidermal layer of the in vitro skin model could be removed similarly to chemical peelings (Fig. 4B, arrows).

**Table 1:** Chemical burning concentrations, time, and washing periods

Tested substance and time	Washing time	Sample *n*
1% phenol, 30 s	10 min in sterilized deionized H_2_O	3
1% phenol, 1 min	10 min in sterilized deionized H_2_O	3
1% phenol, 2 min	10 min in sterilized deionized H_2_O	3
5% phenol, 30 s	10 min in sterilized deionized H_2_O	3
5% phenol, 2 min	10 min in sterilized deionized H_2_O	3
PBS (control), 1 min	10 min on PBS	3

**Fig. 5:**
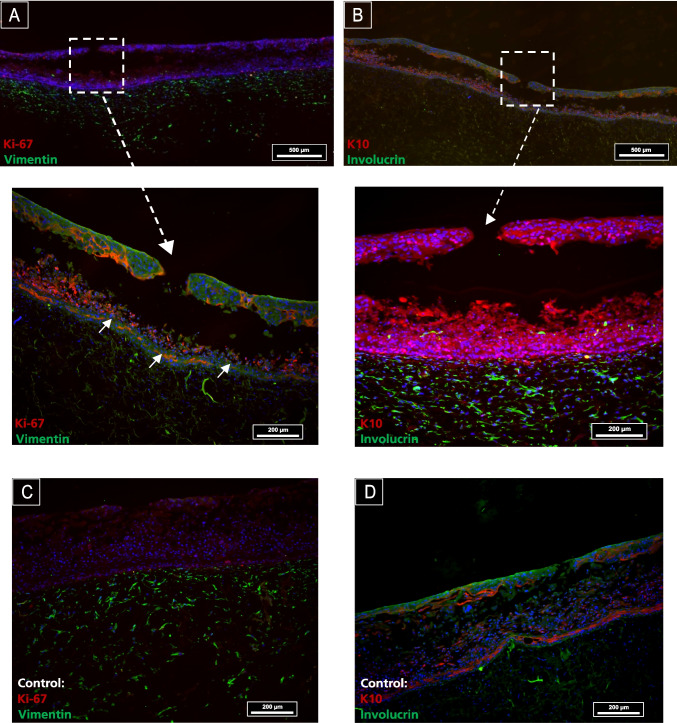
Immunofluorescence analysis of a needle-stamped skin wounding model revealed typical expression patterns across wounded and control tissues. In the wounded area (**A**), Ki-67 and vimentin expression were observed, indicating cellular proliferation and fibroblast phenotype. The wounded region (**B**) exhibited K10 in terminally differentiated keratinocytes and involucrin in cornified keratinocytes. The unwounded control showed baseline Ki-67 and vimentin expression (**C**) alongside the normal distribution of K10 and involucrin in differentiated and cornified keratinocytes, respectively (**D**). The scale bars represent 500 µm (top row) and 200 µm (second and third rows). Staining interpretation: blue = DAPI; red = K10 or Ki-67; green = vimentin or involucrin

In Fig. 5A, it can be seen that the epidermis of the 3D skin model did not detach immediately after 30 s of 5% phenol treatment but rather after 24 h. Therefore, the phenol-wounded tissues were subsequently cultivated for 24 h on the liquid air-lift phase to analyze further layer detachment (Fig. 4B). The diminished cell density in Fig. 5B is further demonstrated by immunohistochemical staining. The control treatment with PBS showed no reaction (Fig. 4C). Chemical treatment presents greater challenges in adaptation as the results are not immediately apparent, and the expansion of the wound largely depends on the catalytical cessation of the chemical reaction. The wound produced by phenol treatment expanded completely across the scaffold and was not clearly defined.

A previous study [22] treated actinic keratosis and Bowen disease skin with 100% phenol, in which phenol completely removed the epidermal layer of treated patients, resulting in the removal of skin tumors after one or two treatments. Additionally, the effectiveness of phenol in treating scarred and wrinkled skin has been compared with laser treatment. The authors concluded that phenol is equally effective as laser treatment in removing scarring and wrinkles, highlighting its significance as a clinical tool [23].

This present study concludes that a 5% phenol treatment for 30 s on bioengineered skin is the fastest and most effective chemical wounding method for entirely removing the epidermal layer. While the resulting wound may not be immediately apparent, it becomes evident by day 1 following the 5% phenol treatment for 30 s. Although this last technique is the most common chemical peeling used in the cosmetic industry, it cannot create a reproducible defined wound since it relies on catalytic cessation after treatment. In contrast to the skin wounding model using stamping needles, the skin wounding model using 5% phenol represents a chemical injury paradigm, offering insights into more complex wound healing scenarios. Phenol treatment induces necrosis of the epidermis and superficial dermis, creating a broader area of tissue damage compared to mechanical models (Fig. 4B). This approach is particularly relevant for studying chemical burns and certain chronic wound conditions. The 5% phenol model allows to examine the effects of chemical-induced tissue destruction on wound healing processes, including the removal of necrotic tissue and the challenges associated with re-epithelialization in the presence of chemical residues [24]. This model is especially useful for investigating the long-term effects of chemical injuries on skin structure and function, as well as for testing treatments aimed at accelerating healing in chemically damaged tissues.

### Functional 3D skin proliferation and differentiation analyzed by immunofluorescence staining

**Fig. 6:**
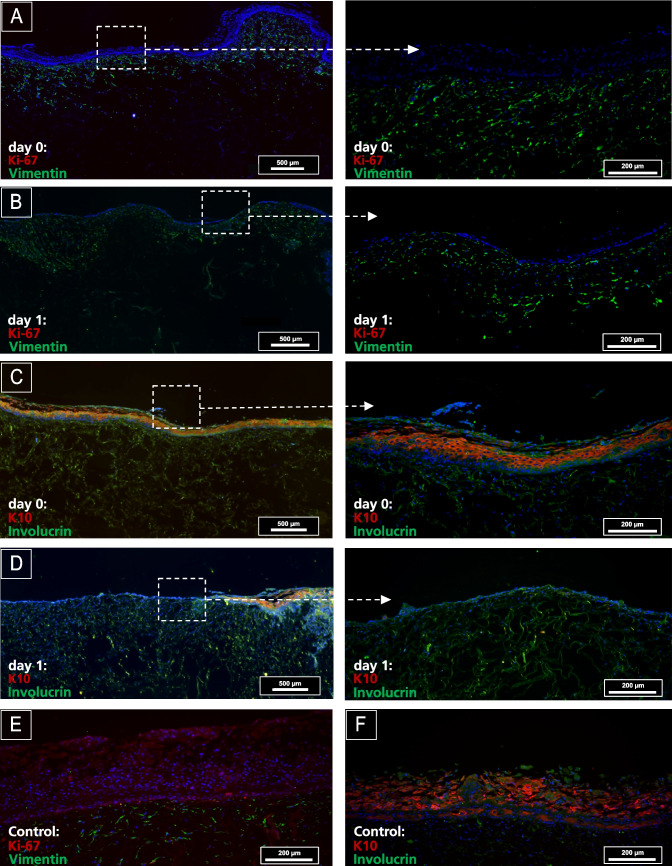
Immunofluorescent labeling demonstrates the expression of Ki-67 expression on control (unwounded) in **E** but absent on day 0 (**A**) and day 1 (**B**) after 5% phenol for 30-s treatment. Detection of K10 in the suprabasal layer, indicative of cell differentiation, was observed in both control samples (**F**) and immediately after wounding day 0 (**C**). However, this expression pattern was absent on day 1 post-wounding (**D**). The scale bars represent 200 µm (right column), 500 µm (left column), and 200 µm for the controls

To visualize the essential properties of the 3D skin wound model and gain critical insight into the functionality of the present 3D skin model, specific proliferation and differentiation markers were immunohistochemically stained and visualized via fluorescence microscopy. The proliferative response before and after wounding was evaluated using Ki-67 as a marker of cell growth. In normal human skin, Ki-67 is dispersed within the nuclei of epidermal keratinocytes, while keratin 10 (K10) is suprabasal differentiation-related keratin specific to the skin and cervix [25]. Immunohistochemistry was performed to compare markers of proliferation (Ki-67) and keratinocyte differentiation (K10) between needle-wounded (Fig. 5) and chemical wounded skin models (Fig. 6), as well as the unwounded control. Actively proliferating keratinocytes cells were visualized by Ki-67 and solid staining in the basal layer of the epidermis in needle wounded samples (Fig. 5A, arrows) and the control (Fig. 5C). The analysis of differentiation and active state of proliferation markers in bioengineered skins models has been vastly discussed and aligns with our results [26, 27]. Other works have focused on the differential keratin expression during the development of a bioengineered skin model. They observed that K10 was fully expressed in the epidermis of the terminally differentiated keratinocytes two weeks after airlifting [28].

In the present study, proliferation was also evident as the differentiated keratinocytes (K10) and protein involucrin moved to the suprabasal layer in needle-wounded 3D skin and control (Fig. 5B and D). The protein involucrin is necessary for forming the cellular envelope in keratinizing epithelia and is detected in granular keratinocytes and upper stratum keratinocytes [29]. To quantify the terminal differentiation of keratinocytes, the population of involucrin-positive cells was visualized on the cornified envelope (Fig. 5B and D). The protein marker vimentin indicates the fibroblast phenotype of cells anchored within the collagen matrix (Fig. 5A and C). The dermal fibroblasts are evenly distributed in both the control and needle-wounded skin models.

For the phenol treatment, the epidermal component exhibited no signs of Ki-67 expression on day 0 and day 1 (Fig. 6A and B) following 5% phenol treatment of 30 s. Detection of K10, indicating cell differentiation, was robust in the suprabasal layer at day 0 but was not evident in the suprabasal layer of the epidermis at day 1 (Fig. 6C and D). Wounding with 5% phenol for 30 s. Entirely detached the epidermis on day 1 (Fig. 6B and D). Vimentin and involucrin were less visible on day 1 after phenol treatment (Fig. 6B and D). The non-wounded control exhibited an intact epidermis with K10-positive proliferating suprabasal keratinocytes and Ki-67 cell proliferation on the basal membrane, respectively (Fig. 6E and F).

Besides proliferation markers, dermal–epidermal junction markers such as collagen IV, laminin V, perlecan, and α6β4 integrin could be helpful tools for skin wound healing studies. Other specific epidermal differentiation markers such as filaggrin, loricrin, and transglutaminase-1 could be investigated in future bioengineered skin wound models. The results demonstrate that the developed 3D skin model closely mimics the in vivo skin situation, accurately representing important biomarkers for skin development. Consequently, it can be employed as a model for wound healing research.

In other studies comparing human skin biological markers with bioengineered skin models, proliferation and differentiation markers have been successfully immunolocalized [30]. Furthermore, our model exhibits a histological architecture and immunolocalization similar to that of human skin.

In this study, our bioengineered skin wound models reveal distinct expression patterns of Ki-67 and K10 between needle-stamped and 5% phenol-induced injuries, highlighting the differential cellular responses to mechanical and chemical injury. Needle-stamped wounds typically exhibit Ki-67 expression in the basal layer near the wound edge, indicating enhanced localized proliferation [31]. In contrast, phenol-induced wounds show no Ki-67 signal, extending beyond the immediate wound edge, likely due to broader tissue damage. Durgun et al. demonstrated that Ki-67 levels can serve as a reliable histological marker for assessing chemical burn depth, with expression levels directly correlating to the severity of chemical injury [32]. Previous studies have demonstrated that K10 expression is initially suppressed or absent in chemically damaged skin areas but gradually recovers as the process of re-epithelialization advances, indicating a progressive restoration of normal epidermal differentiation patterns [33]. In our study, the K10 expression in phenol-induced wounds is significantly reduced or absent initially, compared to needle-stamped wounds that have physiological K10 Expression. These differences manifest in the extent of proliferation, differentiation patterns, and overall wound healing dynamics (Fig. 6C and D). Needle wounds typically heal faster with more rapid re-establishment of normal epidermal structure [34]. It has been reported that in human ex vivo skin subjected to microneedling, histological analysis demonstrated that keratinocytes initiated coverage of the injured area within 6 days post-treatment, indicating a significant progression in the wound healing process [35]. In contrast, phenol wounds may exhibit delayed healing and more pronounced alterations in epidermal marker expression, as seen in Fig. 6B and D. These variations underscore the importance of considering the wound induction method when interpreting results from bioengineered skin models, as cellular responses and healing dynamics can vary significantly between mechanical and chemical injuries.

### Upregulation of IL-1 alpha after wounding

Interleukin 1 alpha (IL-1α) is a crucial proinflammatory cytokine that is constitutively produced and retained in keratinocytes. It is released in response to specific stimuli. Upon wounding, keratinocytes express higher amounts of IL-1α to stimulate the activation and proliferation of neighboring keratinocytes. The released IL-1α serves as a signal to dermal endothelial cells and triggers the activation of dermal fibroblasts [36].

**Fig. 7:**
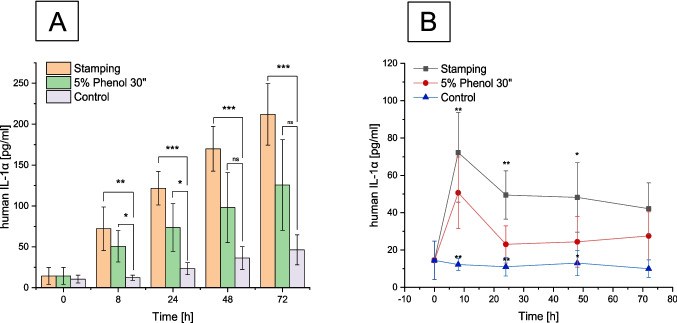
ELISA determination of soluble concentration from IL-1α in cell culture supernatant from wounded 3D skin models with stamping needles (*n* = 3), 5% phenol for 30 s. (*n* = 3), and control (*n* = 3). **A** Cumulative levels of IL-1α of stamping and phenol, including the control group on hours 0, 8, 24, and 48. **B** IL-1α daily levels after injury with stamping needles, 5% phenol 30 s, and unwounded control on hours 0, 8, 24, and 48. Data are mean ± SD in triplicate. Values were significantly different (*p < 0.05, **p < 0.01, and ***p < 0.001) from the control. *NS*, not significant, Bonferroni

For the analysis of human IL-1α, cell culture supernatants were collected from 5% phenol treatment, needle-stamped wounded, and non-wounded (control) 3D skin models. Figure 7A represents the cumulative data of IL-1α expression, while Fig. 7B illustrates the amount of IL-1α expressed over time. For both wounded groups, a constant expression of IL-1α at 70 pg/mL was observed 8 h after needle stamping (^**^p < 0.01) and 50 pg/mL after phenol peeling of the 3D skin models (^*^p < 0.05). The phenol-peeled sample exhibited approximately 50% lower IL-1α expression than the needle-stamped sample at 24 h and 28 h post-injury. It is known that the expression of IL-1α is terminated by an autoregulatory negative feedback mechanism, wherein elevated levels of IL-1α induce the production of its own inhibitors, such as IL-1Ra and soluble IL-1R1 [37, 38]. This mechanism leads to a decrease in IL-1α concentration after 24 h, which correlates with our results. Three days post-wounding, the IL-1α expression decreased to 40 pg/mL after stamp-needle injury and 30 pg/mL after 5% phenol treatment for 30 s. The expression of IL-1α was significantly different (^***^p < 0.001) from the control 72 h after stamp-needle wounding.

The difference in IL-1α expression between the two wounding methods can be correlated to the immunoanalysis of the proliferation marker Ki-67 (Fig. 6A and B). The findings revealed that post-phenol treatment, the epidermis was removed after 24 h, resulting in fewer cells present on the epidermis and a subsequent reduction in IL-1α expression. At 72 h post-wounding, a two-fold decrease in IL-1α expression was recorded for both the needle-injured sample (40 pg/mL) and the sample treated with 5% phenol (30 pg/mL) (Fig. 7B).

The expression of IL-1α for the control was significantly lower with 10 pg/mL at 72 h incubation time (Fig. 7B). Throughout the experiment, the control consistently exhibited a steady expression of 10 pg/mL IL-1α at each time point. This constant expression is attributed to keratinocytes, which retain significant amounts of IL-1α in intracellular stores [39]. Notably, IL-1α was constitutively produced, with secretion increasing to a cumulative total level of 212.5 pg/mL for mechanical needle injury and reaching a maximum cumulative level of 125 pg/mL after phenol exposure 72 h post wounding (Fig. 7A). The non-wounded control expresses a cumulative value of 40 pg/mL IL-1α after 72-h incubation (Fig. 7A).

We could demonstrate that the bioengineered skin displays a graded response to injury, as evidenced by the early production of proinflammatory cytokines such as IL-1α. Previous studies on bioengineered wounding models have assessed cytokine levels after injury by passing the 3D skin model through a mesher [36], with the highest concentration of IL-1α reported 24 h post-injury to the bioengineered skin. Our findings are in line with prior research and literature, confirming a heightened expression of IL-1α within the initial 24 h after wounding [36–38].

Recent studies have elucidated the critical role of IL-1α in wound healing and inflammatory responses. A murine model of incisional wounding demonstrated a strong correlation between IL-1α levels and inflammatory mediator production, underscoring the cytokine ‘s importance in regulating wound healing dynamics [40]. Mechanical injuries, such as tape stripping, have been shown to trigger IL-1α release from keratinocytes. This release activates inflammatory pathways, leading to the production of additional pro-inflammatory cytokines [41].

In summary, the response to a local injury and tissue repair depends significantly on cytokine-mediated cellular activity. Early detection of IL-1α is crucial, as it can signal epidermal damage before tissue damage occurs. Future experiments will focus on evaluating the expression of dermal inflammation markers such as IL-6, a proinflammatory cytokine associated with skin inflammation and healing.

### SEM analysis of fibroblast migration and morphology on collagen scaffolds

**Fig. 8:**
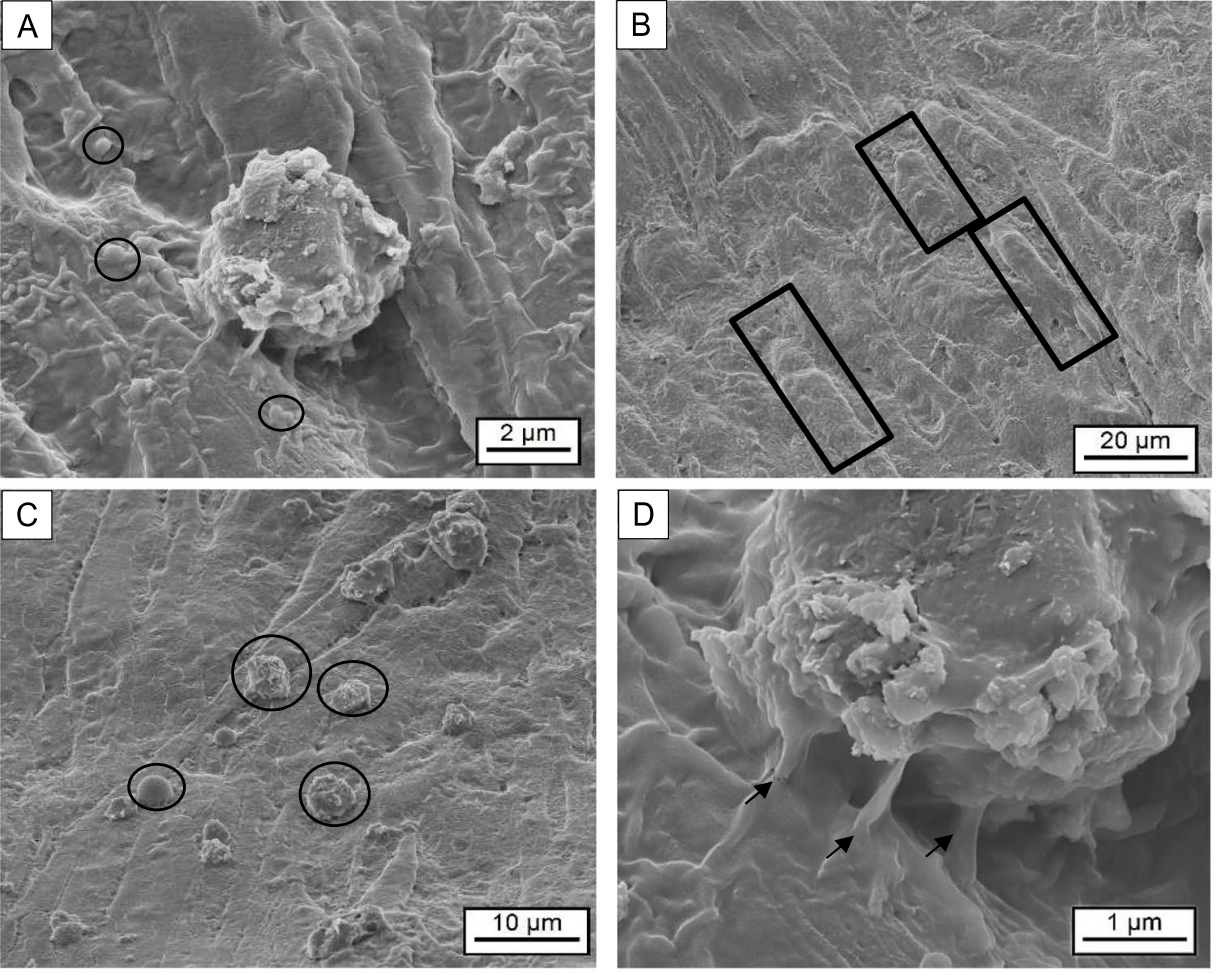
SEM micrographs showing BJ fibroblasts cultured on crosslinked collagen scaffold matrices. **A** The fibroblasts showed a very rough surface containing microvesicles (circles). **B** Filipodia from fibroblasts covering the collagen scaffold surface (squares). **C** Fibroblasts vary in shape (circles). **D** Typical rough surface of a fibroblast and filopodia extensions (arrows)

BJ fibroblasts were cultured in EDC/NHS cross-linked collagen scaffold matrices as described above, and their distribution was studied using SEM images (Fig. 8). After a 3-day incubation period following seeding, a substantial population of fibroblasts became firmly attached to the upper scaffold’s surface. Notably, these fibroblasts displayed a highly textured exterior characterized by the presence of microvesicles (Fig. 8A, circles).

Studies have shown that metalloproteinases are released directly from the fibroblast’s plasma membrane, often taking the form of shedding microvesicles [42]. Fibroblasts extensively envelop the collagen fibers with their cellular surface, giving rise to prominent extensions. (Fig. 8B, squares). In the presence of the collagen scaffold, fibroblasts exhibited different shapes, from round cells to elongated ones (Fig. 8C). The fibroblasts appear to be bound to the collagen fibers, forming filopodia (Fig. 8D, arrows). It has been studied that extensions of filopodia are typical for cells that can alter their actin cytoskeleton as they sense their three-dimensional environment. Furthermore, filopodia contain actin filaments that form focal adhesions. These structures possess the ability to exert forces and attract collagen fragments towards the cell surface of fibroblasts [43].

## Conclusions

This work presents collagen-enriched bioengineered three-dimensional skin wound models within the evolving field of tissue engineering. Progress continues toward developing bioengineered skin models that accurately replicate human skin to assess epidermal-dermal biology in vitro. We believe that consistently reproducible and cost-effective 3D skin wound models could serve as primary models for wound healing research, bridging in vitro and in vivo knowledge.

The developed bioengineered skin wound model incorporates crucial components, arranging cells in a 3D environment similar to the in vivo scenario. This collagen-based model efficiently creates defined wounds while preserving the 3D arrangement of cells, allowing for the expression of typical wound healing biomarkers. This is critical for studying inflammation outcomes, drug screening, cytotoxicity, and stress-related processes affecting skin barrier function.

We report a reproducible epidermal wound in a bioengineered 3D skin model using the stamping-needle wound method. New sharp 250-µm sterile needles were used to generate precise cutaneous wounds, resulting in defined punctures of the epidermis. This method proved to be a fast and cost-effective alternative for creating tailored epidermal wounds.

The second method implemented in this project involved chemical wounding using phenol in different concentrations and incubation times. Treatment with 5% phenol for 30 s proved to be the fastest and most effective method for entirely removing the epidermal layer. Although the effects of this wound were not immediately observed, they became apparent on the first day following phenol treatment. The expansion and corrosive effect of the 5% phenol wound depend entirely on the timing of catalytic stop and chemical washing. While this technique is the most common chemical peeling used in the cosmetic industry, it cannot consistently create a reproducible defined wound.

While our results demonstrate the potential of the 3D skin wounding model for investigating the effects of test substances and wound healing processes, it is important to acknowledge that the designed skin model does not entirely mimic the in vivo situation. This 3D skin wound model can be enhanced by incorporating additional cells such as microvascular endothelial cells, Langerhans’ cells, and melanocytes to create a more human-equivalent skin model. However, we recognize that including these cells in the wounding model will not develop a circulatory or nervous system as in the in vivo scenario. Nonetheless, this approach will facilitate an increased understanding of the interactions between cells that have vital roles in skin biology.

This novel wounding of bioengineered skin creates a wound-like environment that closely resembles real wounds. This allows the study of wound healing processes, testing potential treatments, and understanding the underlying mechanisms involved in wound healing.

The stamping-needle wound method allows for precise control over the wound’s depth, size, and shape by adjusting factors such as needle length, penetration force, and angle. This ensures consistency in the wound models, enabling accurate comparisons and reliable results in subsequent experiments. Additionally, the technique can be easily scaled for high-throughput experiments, making it suitable for large-scale screening of potential wound healing therapeutics or treatments.

Overall, using a stamping needle to perforate a bioengineered skin model to create a “bioengineered skin wound model” offers a controlled, reproducible, standardized, versatile, and scalable approach to studying wound healing processes and evaluating potential treatments.

## Materials and methods

### Collagen scaffold generation and cross-linking

For the collagen homogenization procedure, the collagen flakes obtained from the bovine Achilles tendon (Sigma Aldrich, Germany) were solved in 50 mM acetic acid (VWR Chemicals, Germany) and left overnight at room temperature (RT). Homogenization of the collagen was achieved by dispersing for 2 min at 15,000 rpm (Ultra-Turrax T25, IKA). For the preparation of the collagen scaffolds, 12 cylindric forms (diameter = 1.4 cm, height = 1 cm) were filled with 2.5 mL of homogenized collagen. The filled cylinders were pressed together with screwed Teflon sheets and plates. The cylinders were placed in the ice condenser machine, where the following parameters were applied: freezing at − 35 °C for 4 h at 0.1 bar, thawing for 20 min at − 35 °C, lyophilization at 24 h at 0 °C and 0.1 mbar. The scaffolds were collected the next day from the freeze dryer machine (Ice condenser Alpha 2–4 LSCplus, Martin Christ Gefriertrocknungsanlagen GmbH, Germany) after 24 h of lyophilization. The next day, the collagen scaffolds needed for the first skin model were cross-linked with 10 mM 1-ethyl-3-(3-dimethyl aminopropyl)carbodiimide hydrochloride (EDC) (VWR chemicals, Germany) and 4 mM NHS (N-hydroxysuccinimide) in 75% ethanol. The collagen scaffolds were submerged entirely in the cross-linking solution at RT for 2 h. After incubation, the scaffolds were washed two times with PBS for 5 min and three times with sterile aquadest for 10 min. Following this step, the cross-linked scaffolds were stored at 37 °C in the 5% CO_2_ incubator (Binder GmbH, Germany) overnight until fibroblast seeding.

### Preparation of cells

Primary human epidermal keratinocytes HaCaT cell line from adult human skin (CVCL_0038, DKFZ, Germany) and human foreskin BJ fibroblasts obtained from American Type Culture Collection (CRL-2522, ATCC, USA) were cultured in Dulbecco’s modified Eagle ‘s medium (DMEM; Biowest, Germany) with 10% fetal bovine serum (FBS; Biowest), 1% penicillin–streptomycin (Biowest), 3 mM glutamine (Biowest), and 1% amphotericin B (Biowest). The culture medium was renewed three times per week. Cultures with 70–80% confluency were used to construct the skin model.

### Generation of the 3D skin model

The cross-linked collagen scaffolds were placed into a 12-well plate filled with fibroblast complete medium; DMEM, 10% FBS, 1% penicillin–streptomycin, 1% amphotericin B, 3 mM glutamine, 10 ng/mL human epidermal growth factor (EGF; R&D Systems, UK), and 50 μg/mL ascorbic acid. BJ fibroblasts (7.5 × 10^5^ cells) were seeded onto each collagen scaffold side (area of 1.5394 cm^2^) with *n* = 8. The fibroblasts on the scaffolds were cultured submerged in the fibroblast complete medium. After 2 days, the seeded scaffolds were transferred into a 6-well plate. After 2 weeks, the keratinocytes HaCaT cell line (1 × 10^6^ cells) was inoculated on the collagen scaffolds populated with the BJ fibroblasts. After 50 min of attachment, the cultures were submerged within the complete medium: DMEM, 10% FBS, 1% penicillin–streptomycin, 3 mM glutamine, 1% amphotericin B, 1 ng/mL epithelial growth factor (EGF), 50 μg/mL ascorbic acid for 2 days, and in complete medium supplemented with 1 ng/mL human tumor growth factor alpha (TGF-α; R&D Systems, UK) for the following 7 days. On day 3, the co-culture of BJ fibroblasts and HaCaT keratinocytes were liquid air-lifted. We designed an “air-lifting” platform consisting of a stainless-steel ring (diameter = 1.4 cm) covered with a removable plastic platform (Eppendorf tube cap).

### Generation of the 3D skin wound model by stamping needles and chemical peeling

For the needle stamp wounding, 3D skin models (*n* = 8) were punctured from the top with a stamp containing three needles at a penetration depth of 250 µm. The collected samples were then immediately fixated in 3.7% formaldehyde (Sigma Aldrich) for further processing. Control samples were unpunctured 3D skin models (*n* = 3).

The chemical wounding of the 3D skin models was carried out with phenol (Carl Roth GmbH, Germany) solutions at different concentrations diluted in H_2_O. Thirty microliters of each solution was pipetted on top of the 3D skin model and incubated for defined time spans before rinsing as specified in Table 1. Subsequently, the samples were either immediately fixated in 3.7% formaldehyde or incubated at 37 °C in the 5% CO_2_ incubator for 24 h and then fixated in formaldehyde for further processing.

### Enzyme-linked immunosorbent assay (ELISA) IL-1α

For the human IL-1α analysis, the cell culture supernatant serum was collected from two wounded sample groups and controls in triplicates (Table 2). The medium was compiled entirely from each 3D skin model well and replaced with a new medium every 24 h. The collected medium was centrifuged at 2000 × *g* for 5 min at 4 °C, and the supernatant was stored at − 80 °C until the ELISA assay was carried out.

**Table 2:** 3D skin models (*n* = 9) used for ELISA

Sample	Supernatant collection times in h	Sample *n*
Needle stamped	0, 8, 24, 48, and 72	3
5% phenol for 30 s	0, 8, 24, 48, and 72	3
Control (no wounding)	0, 8, 24, 48, and 72	3

The ELISA was carried out according to the manufacturer’s manual. A pre-coated human IL-1α 96-well microplate (R&D Systems, UK) was incubated with 50 µL of assay diluent. Two hundred microliters of standards (250 pg/mL), diluent buffer for control, and cell culture supernatants from the 3D wounded skin model (Table 2) were incubated for 2 h at room temperature. After this step, the microplate was washed three times with wash buffer PBST (0.05% Tween 20 in PBS, pH 7.2–7.4). The microplate was incubated for 1 h with 200 µL of human IL-1α HRP-conjugate in each well. The washing step was repeated after incubation.

Next, 200 µL of substrate solution (tetramethylbenzidine diluted 1:2 in hydrogen peroxide) was incubated for 20 min at room temperature. Fifty microliters of stop solution (2N sulfuric acid H_2_SO_4_) was immediately added to each well to stop the enzyme reaction. The samples were mixed thoroughly before each reading. The optical density (O.D.) absorbance was measured spectrophotometrically at 570 nm in a microplate reader FLUOstar Omega (BMG Lab Technologies GmbH, Germany), and then the concentration of human IL-1α was calculated.

### Cell titer-blue cell vitality

Effects on cell vitality and apoptosis were assessed using Cell Titer Blue (Promega, USA) according to the manufacturer’s manual. The cross-linked collagen scaffolds’ upper sides were seeded with BJ fibroblasts (1.3 × 10^5^ cells per scaffold), vitality 99%, *n* = 6. The fibroblasts on the scaffolds were cultured submerged in the complete medium (DMEM with 10% FBS, 1% penicillin–streptomycin, 3 mM glutamine, 1% amphotericin B). After 2 days, the seeded scaffolds were transferred to a 6-well plate. The measurements of cell activity after incubation for 8, 24, 36, 48, and 72 h were performed as separate experiments. For control, cross-linked collagen scaffolds without cells were used (*n* = 3). 500 µL of the Cell Titer-Blue mixture was added to the 4,500 µL complete medium. The Cell Titer-Blue mixture was pipetted onto the scaffolds seeded with cells as well as the controls. All samples were incubated for 2 h (37 °C, 5% CO_2_) to assess the fibroblasts’ ability to reduce resazurin into a fluorescent end product resorufin. The collected samples from each well (100 µL) were transferred to a dark 96-well reading plate (BRAND, Germany). Relative fluorescence was measured using a Lumistar Omega (excitation at 544 nm and emission at 590 nm; BMG Lab Technologies GmbH) using the Lumistar Omega software. The results were compared to the cell culture media of negative controls (scaffolds without cells) and adjusted for background signal. The experiment was performed in triplicates and repeated twice.

### CellTiter-Glo 3D cell viability assay

In a 12-well plate, the cross-linked collagen scaffolds’ upper sides (area = 1.5 cm^2^) were seeded with BJ fibroblasts (1.3 × 10^5^ cells per scaffold), vitality 98%, *n* = 6. The fibroblasts on the scaffolds were cultured while submerged in a complete medium (DMEM with 10% FBS, 1% penicillin–streptomycin, 3 µm glutamine, 1% amphotericin B). The cell medium was changed every 24 h. After 2 days, the seeded scaffolds were transferred to a 6-well plate. The cells were assessed for ATP content according to the manufacturer’s manual on day 1, day 2, and day 3 with the Cell‐Titer Glo 3D viability assay (Promega, USA) as separate experiments. As controls, cross-linked collagen scaffolds without cells were used (*n* = 3). In a 24-well plate, the transferred seeded scaffolds and controls were incubated for 30 min while being vigorously shaken and pipetted with 300 µL of the CellTiter-Glo 3D reagent to induce cell lysis. In a dark 96-well plate, 50 µL of the CellTiter-Glo 3D reagent was given to each well. After this, 100 µL of the cell lysis, control, and standards were transferred to each well. The luminescent signal was recorded with the FLUOstar. The samples’ luminescence was compared to the luminescence of ATP standard curve to determine ATP concentration detected by CellTiter-Glo 3D reagent in the samples. The experiment was performed in triplicates and repeated twice.

### Histochemical analysis by H&E staining of 3D wounded skin tissue sections

After performing the 3D skin wounding procedures, the samples were immersed in a 3.7% formaldehyde solution overnight. Following fixation, the samples were dissected to select appropriate areas for examination. After this, the specimens were placed in suitably labeled cassettes. The dehydration process involved immersion in progressively increasing concentrations of 50%, 70%, 90%, and 95% ethanol. The samples were then fixated overnight in isopropanol to avoid excessive distortion of the 3D wounded model. The next day, xylol (Carl Roth GmbH) was used for clearing the ethanol from the tissue samples. The xylol solvent was replaced by molten paraffin wax. The skin model samples were sectioned to a thickness of 7 using an HM 355 rotary microtome.

Before staining, all materials were allowed to equilibrate at room temperature, and the reagents were prepared according to the manufacturer’s instructions (Carl Roth GmbH). The procedure commenced with deparaffinization of the samples using xylol, followed by sequential immersion in graded ethanol concentrations of 100%, 70%, and 50%. Subsequently, formalin-fixed 3D skin models were rehydrated with distilled water. The slides were stained with combined hematoxylin (Carl Roth GmbH), Weigert’s Iron, and eosin G 0.1% (Carl Roth GmbH) water staining, followed by washing steps with aqua dest. After staining, the slides were mounted with Roti-Histokitt (Carl Roth GmbH) and covered with cover glasses 24 × 60 mm^2^ until the light microscopic analysis (Carl Zeiss, Axiovert 25 with HBO 50 lamp) was carried out.

### Immunostaining on 3D wounded skin tissue sections

The paraffinized sample sections were mounted onto microscope slides (VWR). They underwent deparaffinization and rehydration using xylol, followed by sequential immersion in graded ethanol concentrations of 100%, 96%, 80%, and 70%. A heat-induced epitope retrieval step is conducted to facilitate access to cross-linked epitopes created by formaldehyde. The hydrated slides were submerged in epitope retrieval Tris–EDTA buffer; 10 mM Tris base (Carl Roth GmbH), 1 mM EDTA solution (Sigma Aldrich GmbH), 0.05% Tween 20 (Carl Roth GmbH), pH 9.0; and heated to boil for 20 min in a microwave at 850 W. The slides were allowed to cool for 10 min in aqua dest. After that, the immunohistochemical staining was performed. The antigen retrieved slides were blocked for 1.5 h in blocking solution; 2% goat serum (Abcam) in 2% BSA/PBS (Thermo Fisher). The samples were then incubated with the primary antibody cocktail anti-vimentin FITC conjugate (ProGen; 4 µg/mL), and anti-Ki67 (Abcam; 1 µg/mL) diluted in blocking solution for staining 1 and anti-involucrin antibody (Abcam; 1 µg/mL) with anti-cytokeratin 10 (Abcam; 1.4 µg/mL) for staining 2. After 1 h of incubation, the sections were washed with PBS/0.01% Tween 20 and incubated for 1 h with the secondary antibodies Alexa flour 647 (Abcam; 1.6 µg/ml), and DAPI (Carl Roth; 1 µg/mL), diluted in blocking solution for staining 1 and Alexa Fluor 647 (1.6 µg/mL), Alexa Fluor 488 (Abcam; 1.6 µg/mL), and DAPI (0.1 µg/mL) for staining 2. The samples were washed with PBS/0.01% Tween 20 and then submerged for 5 min in PBS solution. The tissue sections were mounted with fluorescence mounting medium (Sigma Aldrich) and stored at − 20 °C until fluorescence microscopic analysis (Keyence, BZ-X810).

### Scanning electron microscopy (SEM)

The collagen scaffolds were cross-linked with 10 mM EDC and 4 mM NHS (N-hydroxy-succinimide) in 75% ethanol for 2 h at room temperature (*n* = 2). The top sides of the cross-linked scaffolds were seeded with 10 × 10^4^ cells, vitality 98.47%. SEM was employed to observe high-resolution features of fibroblasts BJ grown on the collagen scaffolds (*n* = 3). After 3 days of incubation, all three samples were washed with 1 × PBS and fixed with 2% w/v glutardialdehyde (Biowest) in 1 × PBS for 2 h. After fixation, the specimens were washed twice with PBS for 10 min each wash. The samples were fixated again in 2% osmium tetroxide (Sigma Aldrich) in PBS for 45 min. Then, the samples were dehydrated in a graded series of ethanol (10%, 30%, 50%, 70%, 90%, and 100%) for 20 min each and left in 100% water-free ethanol for 24 h at RT. Next, the samples were dried with 30%, 50%, and 60% of hexamethyldisilazane (HMDS; Sigma Aldrich) mixed with 100% water-free ethanol for 1 h and only HMDS for 20 min. Finally, the dried samples were glued to disk plates with Leit-C conductive carbon (Plano, Germany). The surface of the dried samples was metalized (2–3 nm) by sputter coating with platinum. The morphology of the adherent fibroblast cells on the cross-linked scaffolds was observed by SEM using a Quanta 3D FEG (FEI/Thermo Fisher).

## Statistical analysis

Statistical analysis was performed using Origin 2019b software, and statistical significance was considered when *p* < 0.05. Bonferroni statistic test was used for specific experiments. In the depicted graphics, we used * to denote *p*-values, in which ^*^p < 0.05, ^**^p < 0.01, and ^***^p < 0.001.

## Data Availability

All data are available in the main text.
